# The Chromatin Remodeling Factor CSB Recruits Histone Acetyltransferase PCAF to rRNA Gene Promoters in Active State for Transcription Initiation

**DOI:** 10.1371/journal.pone.0062668

**Published:** 2013-05-07

**Authors:** Meili Shen, Tingting Zhou, Wenbing Xie, Te Ling, Qiaoyun Zhu, Le Zong, Guoliang Lyu, Qianqian Gao, Feixiong Zhang, Wei Tao

**Affiliations:** 1 Key Laboratory of Cell Proliferation and Differentiation, National Key Laboratory of Protein Engineering and Plant Gene Engineering, College of Life Science, Peking University, Beijing, China; 2 College of Life Science, Capital Normal University, Beijing, China; Texas A&M University, United States of America

## Abstract

The promoters of poised rRNA genes (rDNA) are marked by both euchromatic and heterochromatic histone modifications and are associated with two transcription factors, UBF and SL1 that nucleate transcription complex formation. Active rRNA genes contain only euchromatic histone modifications and are loaded with all components of transcriptional initiation complex including RNA polymerase I. Coupled with histone acetylation and RNA polymerase I targeting, poised promoters can be converted to active ones by ATP-dependent chromatin remodeling factor CSB for initiation of rDNA transcription. However, it is not clear how dynamic histone modifications induce the assembly of polymerase I transcription initiation complex to active promoters during such conversion. Here we show that a complex consisting of CSB, RNA polymerase I and histone acetyltransferase PCAF is present at the rDNA promoters in active state. CSB is required for the association of PCAF with rDNA, which induces acetylation of histone H4 and histone H3K9. Overexpression of CSB promotes the association of PCAF with rDNA. Knockdown of PCAF leads to decreased levels of H4ac and H3K9ac at rDNA promoters, prevents the association of RNA polymerase I and inhibits pre-rRNA synthesis. The results demonstrate that CSB recruits PCAF to rDNA, which allows histone acetylation that is required for the assembly of polymerase I transcription initiation complex during the transition from poised to active state of rRNA genes, suggesting that CSB and PCAF play cooperative roles to establish the active state of rRNA genes by histone acetylation.

## Introduction

Ribosomal genes are transcribed by transcription machinery of RNA polymerase I (Pol I). Transcription rate of rDNA is altered by reversible phosphorylation and acetylation of Pol I transcription factors that affect the efficiency of transcription initiation, or dynamic epigenetic modifications that alter the ratio of active to silent copies of rRNA genes, thereby adjusting the number of genes that are involved in active transcription [1]–[4]. Recent studies have shown that rRNA genes exist in three different epigenetic states [4], [5]. Active promoters are unmethylated and exhibit euchromatic features, silent promoters are methylated and exhibit heterochromatic histone modifications, and poised promoters are unmethylated and marked by hypoacetylated histone H4 and trimethylated H3K4 (H3K4me3) [5]. Initiation of rRNA gene transcription requires the conversion from poised configuration to active state that is controlled by the chromatin remodeler protein, CSB (Cockayne syndrome group B).

CSB is an ATP-dependent chromatin remodeler protein that belongs to the SWI2/SNF2 family [6]–[8]. Defects in CSB gene can cause Cockayne syndrome, which is a rare autosomal recessive disorder [9], [10]. CSB plays a role in the transcriptional regulation of all three classes of nuclear RNA polymerases [11]–[13]. As a member of the complex comprising Pol I, TFIIH and basal Pol I transcription factors [14], CSB interacts with histone methyltransferase G9a, which methylates histone H3 on lysine 9 (H3K9me2) in the pre-rRNA coding region thus facilitating transcription elongation [15].

On the rDNA promoters, CSB is able to shift nucleosomes from poised to active positions [5]. During the shift, assembly of Pol initiation complex is induced, resulting in the start of rDNA transcription. In addition to nucleosomal positions, histone modifications on poised promoters are also very different from that on active promoters. For example, histones on promoters in active state are acetylated while those on promoters in poised state are not. Thus it is conceivable that there could be changes in histone modifications associated with the nucleosomal shifting to facilitate the assembly of Pol I initiation complex. However, it is not clear how changes in histone modifications that occur during the conversion of promoters from poised to active state induce the assembly of Pol I transcription initiation complex.

Histone acetylation plays an important role in modulating chromatin structure and function [16]. There are two classes of histone modifying enzymes with antagonistic effects that control the acetylation state of a given chromatin: histone acetyltransferases (HATs) and histone deacetylases (HDACs). In general, acetylation is linked to transcriptional activation, and HATs have been identified as transcriptional co-activators. HATs are divided into five families including GCN5-related N-acetyltransferases (GNATs), MYST (for MOZ, Ybf2/Sas3, Sas2 and Tip60)-related HATs, p300/CBP HATs, general transcription factor HATs such as the TFIID subunit TAF250, and nuclear hormone-related HATs such as SRC1 and ACTR (SRC3) [17], [18]. PCAF (p300/CBP associated factor) belongs to the GNATs family and is enriched in promoters of active genes [19]. PCAF contains two functional domains including an N-terminal acetyltransferase domain (HAT) and a C-terminal bromodomain that is believed to interact with acetyl-lysine residue. PCAF can acetylate histones and other proteins involved in transcription [20], [21].

In this study, we have determined that CSB interacts with PCAF and recruits PCAF to rDNA promoter region. In active nucleosomes, PCAF induces acetylation of histone H4 and H3K9, and thereafter the recruitment of Polymerase I to rDNA promoters, resulting in the start of rDNA transcription. These results demonstrate a novel mechanism for rDNA transcription initiation mediated by PCAF and CSB.

## Results

### CSB Interacts with PCAF

**Figure 1:**
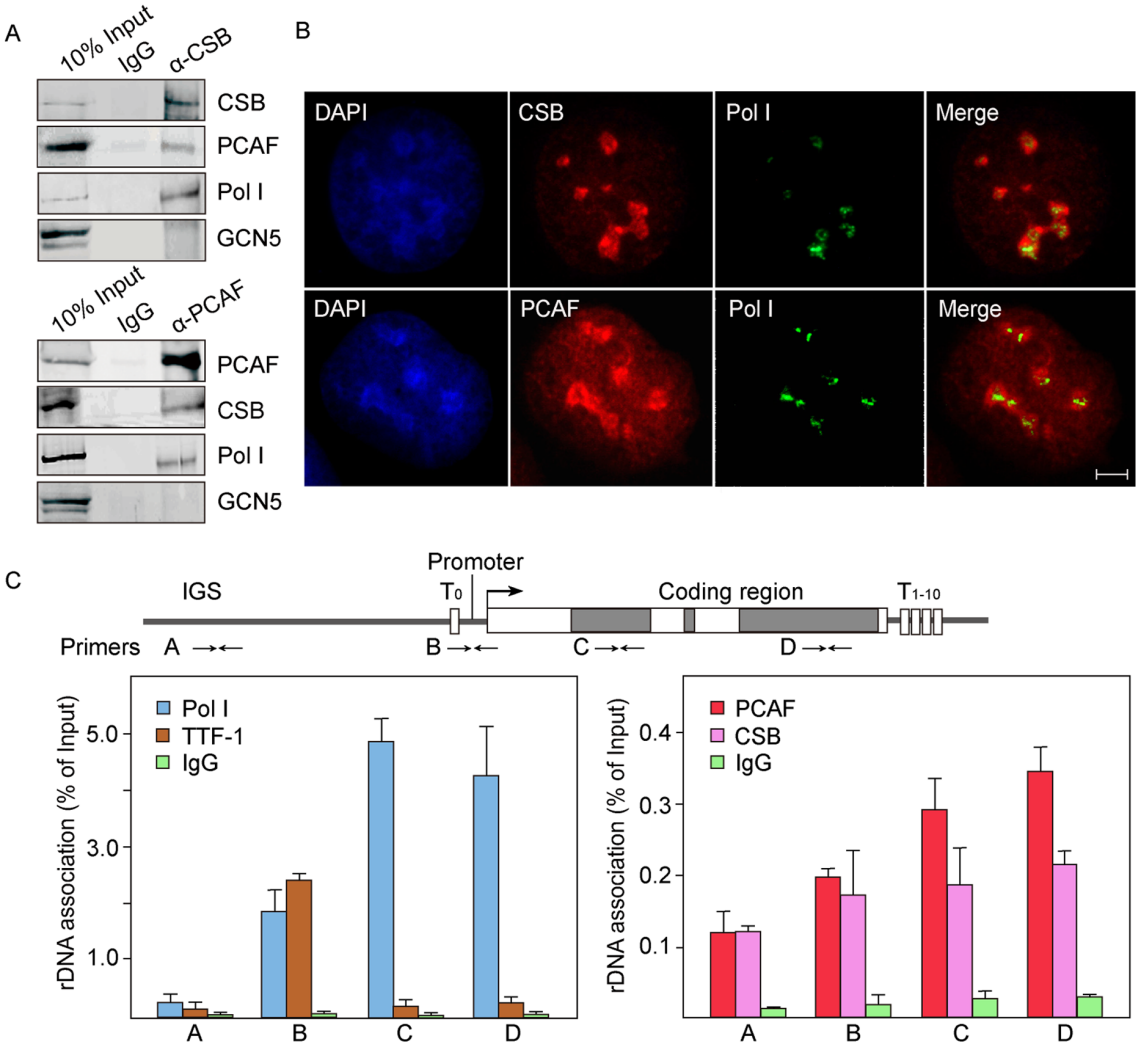
PCAF interacts with CSB and Pol I. A. PCAF interacts with CSB and Pol I *in vivo*. Nuclear extracts from 293T cells were incubated with control IgGs, anti-CSB or anti-PCAF antibodies. About 10% of input and 80% of precipitated proteins were analyzed on western blots. B. CSB, PCAF and Pol I co-localize in nucleoli. Indirect immunofluorescence shows the localization of CSB, PCAF and Pol I in U2OS cells. Scale bar, 5 µm. C. CSB and PCAF are associated with rDNA. Cross-linked chromatins from NIH 3T3 cells were incubated with antibodies against IgG, Pol I (RPA194), TTF-1, CSB and PCAF. The precipitated DNA was analyzed by qRT-PCR using primer pairs that amplify the indicated regions in mouse rDNA. Primer A: intergenic spacer (IGS), primer B: promoter, primer C: 18S coding region, primer D: 28S coding region. Values of the average %IP (±standard deviation) for specific antibodies normalized to input DNA are shown on the bar graph. Positions of the upstream terminator T_0_, rDNA promoter, pre-rRNA coding region and downstream terminators (T_1–10_) are indicated in the scheme above. Error bars represent standard deviation of three independent experiments.

CSB is capable of mediating the transition of poised rDNA promoters to active state [5]. Considering that poised rDNA promoters are histone hypoacetylated and active promoters are hyperacetylated, we reasoned that CSB could recruit histone acetyltransferases for the switch of rDNA promoters from poised to active state. We then identified the specific type of histone acetyltransferase that interacted with CSB by performing co-immunoprecipitation experiments. We found that CSB interacted with histone acetyltransferase PCAF as well as RNA polymerase I, but not with GCN5 in both human 293T and mouse NIH 3T3 cells (Figure 1A, Figure S1).

To assess the biological function of PCAF for rDNA transcription, we investigated its cellular localization using indirect immunofluorescence assay. Results showed the partial labeling of endogenous PCAF co-localized with Pol I in nucleoli (Figure 1B). To investigate the association between PCAF and rDNA, we designed primers from different regions of the rDNA repeats and determined the occupancy of PCAF, TTF1, CSB and Pol I by chromatin immunoprecipitation (ChIP). The results indicated the association of Pol I with rDNA promoter and pre-rRNA coding region, whereas the binding of TTF-1 was restricted to the rDNA promoter. PCAF co-localized with CSB, occupying the rDNA promoter and pre-rRNA coding region (Figure 1C, Table S2). These findings suggested that PCAF may play a role in rDNA transcription.

### CSB Recruits PCAF to rDNA and Facilitates the Interaction of PCAF with Pol I

**Figure 2:**
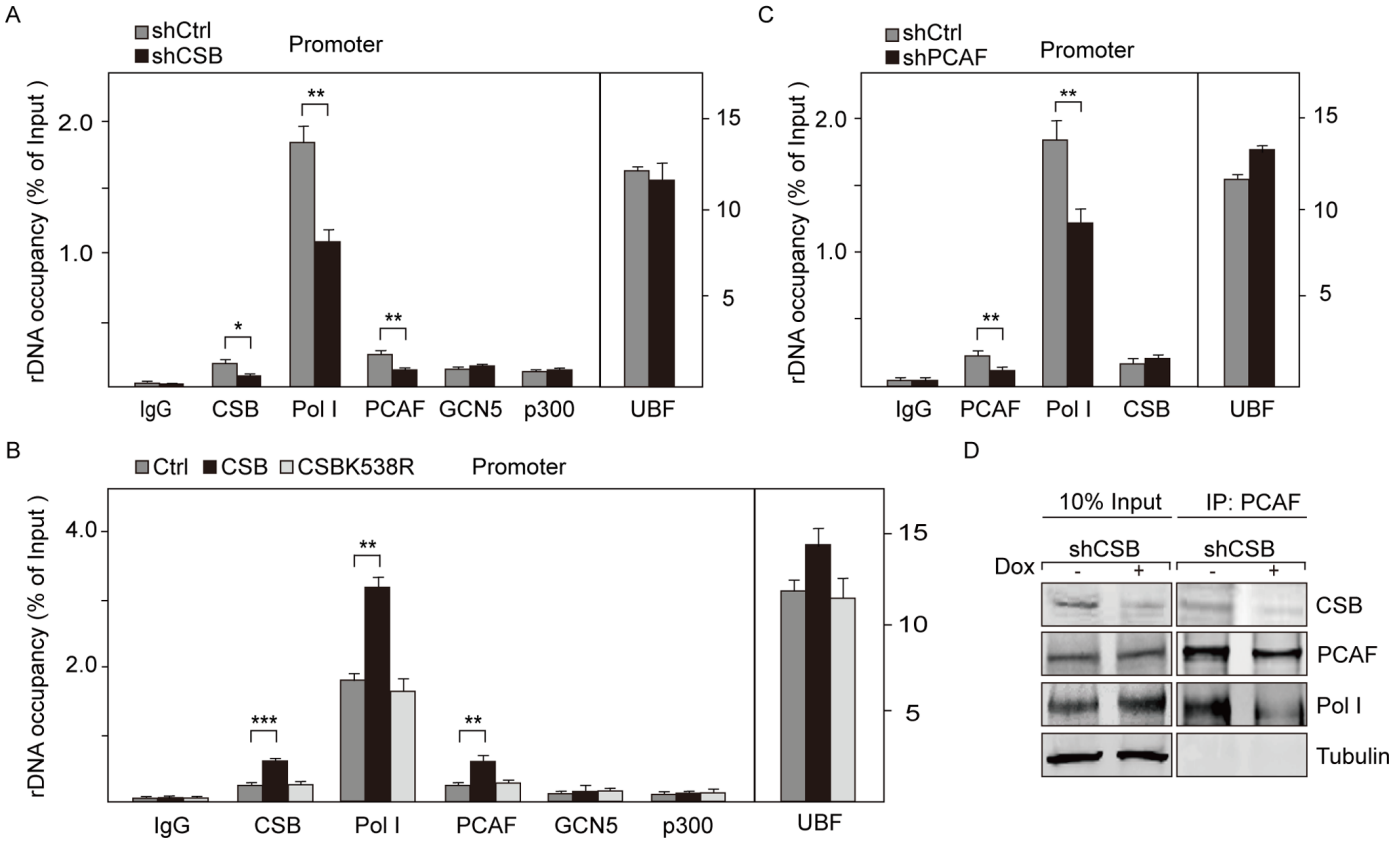
CSB recruits PCAF to rDNA promoter. A. Inducible knockdown of CSB impairs Pol I and PCAF occupancy at rDNA promoter. Knockdown of CSB by CSB-specific shRNA (shCSB) was induced with tetracycline (doxycycline-treated, 1 µg/ml, 72 hr). qRT-PCR data show the associations of CSB, UBF, Pol I, PCAF, GCN5 and p300 with the rDNA promoter in CSB knockdown cells (shCSB) and control cells without treatment with tetracycline (shCtrl). The immunoprecipitated DNA with specific antibodies was normalized to input DNA. Values of the average %IP (±standard deviation) for specific antibodies are shown on the bar graph with the standard deviations from three independent experiments. *P value <0.05, **P value <0.01. B. Overexpression of CSB increases the associations of Pol I and PCAF with rDNA promoter. ChIP data were from NIH 3T3 cells infected with retroviruses encoding wildtype or mutant CSB. The immunoprecipitated DNA from CSB overexpressed cells and mock-transfected cells (Ctrl) was normalized to input DNA. The levels of indicated proteins are shown with the standard deviation from three independent experiments. **P value <0.01, ***P value <0.001. C. Knockdown of PCAF decreases the binding of Pol I, but not UBF and CSB to the rDNA promoter. ChIP assays show the associations of UBF, Pol I, PCAF and CSB with rDNA promoter after knockdown of PCAF in NIH 3T3 cells by PCAF-specific shRNA. The immunoprecipitated DNA with specific antibodies from PCAF knockdown cells (shPCAF) and control cells (shCtrl) was normalized to input DNA. Values of the average %IP (±standard deviation) are shown on the bar graph with the standard deviations from three independent experiments. **P value <0.01. D. Association of PCAF with Pol I depends on CSB. Nuclear extracts from NIH 3T3 cells transfected with either control duplex shRNA or CSB-specific shRNA were incubated with anti-PCAF antibodies or control IgGs. About 10% of input and 80% of precipitated proteins were analyzed on western blots.

Because CSB interacted with PCAF, we reasoned that CSB might target PCAF to rDNA promoters. Indeed, even though all three histone acetyltransferases (PCAF, p300 and GCN5) were previously shown to be associated with rDNA [22]–[24], CSB knockdown reduced the binding of PCAF to rDNA promoter and coding region without affecting GCN5 and p300 binding (Figure 2A, Figure S2A). Consistently, overexpression of wildtype CSB increased the association of PCAF with rDNA while overexpression of ATPase-deficient mutant (CSBK538R) did not affect PCAF occupancy (Figure 2B, Figure S2B). Significantly, knockdown of PCAF did not decrease rDNA occupancy of CSB (Figure 2C, Figure S2C, Figure S2D), whereas depletion of CSB abolished the association of PCAF with Pol I (Figure 2D). In addition, knockdown of PCAF did not affect the association of CSB with Pol I (Figure S2E), suggesting that the interaction of PCAF with Pol I depended on CSB. Thus, knockdown of CSB disassociated PCAF from rDNA and disrupted the interaction between PCAF and Pol I at the rDNA locus. Therefore, CSB is capable of targeting PCAF to rDNA to facilitate the association of PCAF with Pol I.

### PCAF Localizes with Pol I on rDNA Promoters in Active State

**Figure 3:**
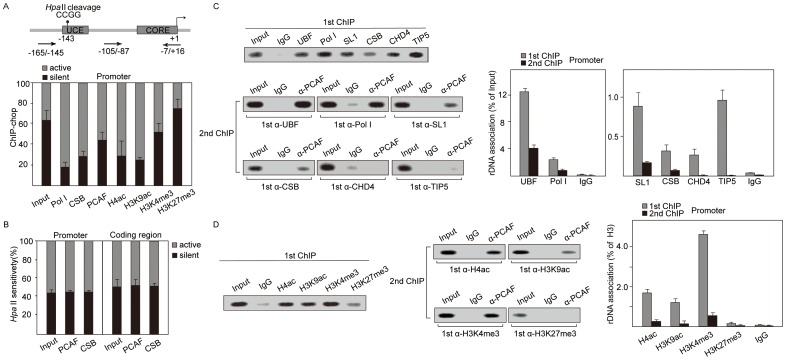
PCAF binds to rDNA promoters in active state. A. PCAF tends to be associated with unmethylated rRNA genes. Cross-linked chromatin from NIH 3T3 cells was precipitated with the indicated antibodies and digested with *Hpa* II before PCR amplification. Relative levels of *Hpa* II-resistant, inactive rDNA copies (black bars) and unmethylated, active copies (gray bars) were determined by qRT-PCR using primer pairs flanking the *Hpa* II sites on the rDNA promoter. Error bars represent standard deviation (n = 3). B. Overexpression of PCAF or CSB does not affect rDNA methylation status. qRT-PCR data show the levels of *Hpa* II-resistant, inactive genes (black bars) and *Hpa* II-sensitive, active genes (gray bars) in NIH 3T3 cells overexpressing PCAF, CSB and mock-transfected cells (Input). Error bars represent standard deviation (n = 3). C. PCAF occupies the same rDNA promoter sequences with Pol I. Cross-linked chromatins were immunoprecipitated with antibodies against UBF, Pol I, SL1, CSB, CHD4 and TIP5 (1st ChIP), followed by precipitation with antibodies against PCAF (2nd ChIP). Co-precipitated DNA was analyzed by PCR and quantified by qPCR using primer pair B shown in Fig. 1C. Values of the average %IP (±standard deviation) for indicated proteins normalized to input DNA are shown on the bar graph (n = 3). 20% of input is shown. D. PCAF co-localizes with H4ac, H3K9ac and H3K4me3. Cross-linked chromatins were immunoprecipitated with antibodies against indicated histone antibodies (1st ChIP) before precipitation with PCAF (2nd ChIP). Co-precipitated DNA was analyzed by PCR and quantified by qPCR using primer pair B shown in Fig. 1C. Values of the average %IP (±standard deviation) for indicated histone modifications normalized to H3 are shown on the bar graph (n = 3). 20% of input is shown.

The rDNA promoters in active and poised states are unmethylated and sensitive to *Hpa* II digestion, while methylated rDNA promoters in silent state are resistant to *Hpa* II cleavage [25]. To determine the occupancy of PCAF on methylated or unmethylated rDNA promoters, we assayed the methylation state of PCAF-associated rDNA by chromatin immunoprecipitation (ChIP)-chop assays. The results showed that PCAF, CSB, H4ac and H3K9ac were all preferentially associated with unmethylated *Hpa* II-sensitive rDNA (Figure 3A, Figure S3A). Furthermore, *Hpa* II digestion showed that overexpression of PCAF and CSB did not affect rDNA methylation status (Figure 3B). These results suggested that PCAF tends to associate with active or poised promoters of rDNA that are unmethylated.

To further determine whether PCAF localized at poised or active rDNA promoters, we performed sequential ChIP assays using antibodies against UBF, Pol I, SLl, CSB, CHD4 and TIP5 followed by immunoprecipitation of PCAF. The results showed that PCAF co-precipitated with UBF, Pol I, SL1 and CSB, but not with TIP5 and CHD4 (Figure 3C). Considering that Pol I exclusively localized on active promoters, while TIP5 and CHD4 exclusively on silent promoters or poised promoters, respectively [5], our results indicated that PCAF is associated with active promoters along with CSB and Pol I transcriptional machinery.

More importantly, sequential ChIP revealed co-localization of PCAF with H4ac, H3K9ac and H3K4me3, which were active histone modifications and were markers of rDNA promoters in active state, whereas PCAF did not localize with H3K27me3 (Figure 3D) that occurred on poised promoters, emphasizing that PCAF associates with rDNA promoters in active state.

### HAT and Bromodomain of PCAF are Required for the Association of Pol I with rDNA Promoter

**Figure 4:**
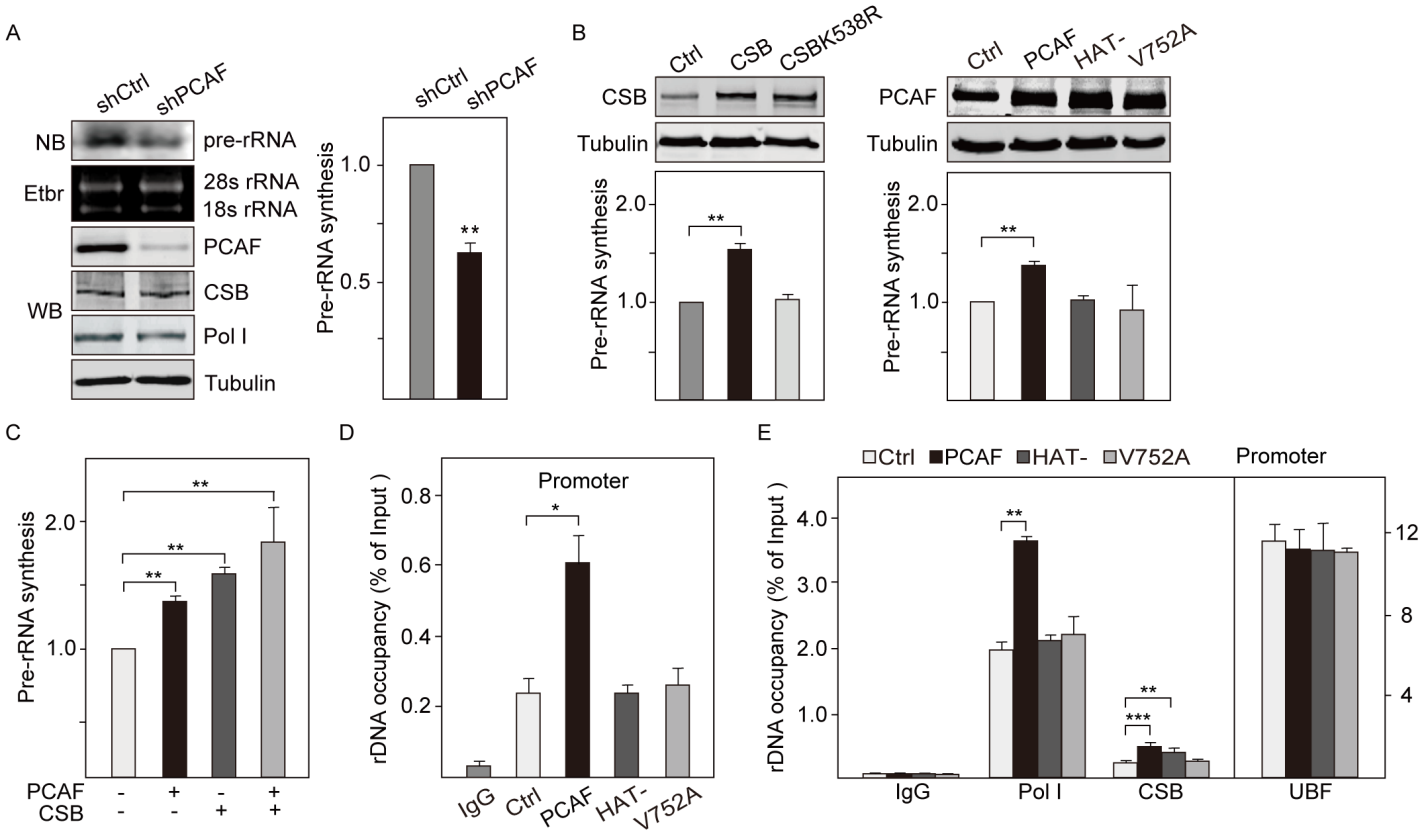
PCAF targets Pol I to rDNA promoters. A. Depletion of PCAF impairs 45S pre-rRNA synthesis. Northern blot (NB) shows the level of pre-rRNA in NIH 3T3 cells infected with lentiviruses encoding PCAF-specific shRNA (shPCAF) or control shRNA (shCtrl). Ethidium bromide (EtBr) stained cellular rRNA. Knockdown of PCAF was determined by western blots using antibodies against PCAF, CSB, Pol I and tubulin. 45S pre-rRNA synthesis was measured by qRT-PCR and normalized to GAPDH mRNA (n = 3). **P value <0.01. B. Overexpression of CSB or PCAF stimulates Pol I transcription. Wildtype or mutant CSB and PCAF were overexpressed in NIH 3T3 cells by retroviral infection and cells were harvested after 8 days. The control assays were done in mock infected cells (Ctrl). 45S pre-rRNA levels normalized to GAPDH mRNA were quantified by qRT-PCR (n = 3). **P value <0.01. Western blots show the level of CSB or PCAF overexpression (top). C. PCAF and CSB cooperate in Pol I transcription activation. NIH 3T3 cells were infected with retroviruses encoding PCAF and CSB, and 45S pre-rRNA levels normalized to GAPDH mRNA were monitored by qRT-PCR (n = 3). **P value <0.01. D. PCAF mutants do not bind to rDNA promoter. ChIP data showing the occupancy of PCAF and PCAF mutants on rDNA promoters in NIH 3T3 cells upon overexpression of wildtype PCAF, HAT-deficient mutant and bromodomain mutant. The immunoprecipitated DNA from PCAF overexpressed cells and mock-transfected cells (Ctrl) was normalized to input DNA. The levels of indicated proteins are shown with the standard deviation from three independent experiments. *P value <0.05. E. sOverexpression of PCAF increases the occupancy of Pol I and CSB on rDNA promoter. ChIP data show the occupancy of UBF, Pol I and CSB on rDNA promoter in NIH 3T3 cells overexpressing wildtype PCAF, HAT-deficient mutant or bromodomain mutant. Values of the average %IP (±standard deviation) for indicated proteins from PCAF overexpressed cells and mock-transfected cells (Ctrl) were normalized to input DNA. Error bars represent standard deviation of three independent experiments. **P value <0.01, ***P value <0.001.

To determine the role of PCAF in rDNA transcription, we measured the levels of pre-rRNA synthesis in PCAF and CSB knockdown cells by northern blot and qRT-PCR. The results showed a decrease in the level of pre-rRNA synthesis after PCAF and CSB knockdown (Figure 4A, Figure S4A). Notably, pulse labeling with fluorouridine incorporation showed reduced level of rDNA transcription upon depletion of CSB and PCAF (Figure S4B),supporting previous results that overexpression of PCAF and CSB stimulated rDNA transcription [15], [22]. However, depletion of PCAF did not affect the expression levels of Pol I transcription machinery components and CSB (Figure 4A, Figure S4A), indicating that PCAF is required for efficient rDNA transcription.

To determine the functional domain of PCAF in rDNA transcription, we generated two PCAF mutant proteins. One had substitutions in two conserved residues (Y616A/Y617A) in the HAT domain, which disrupted the intrinsic HAT activity [26]. The second mutation was in the bromodomain (V752A), which abolished the interaction with acetyl-lysine residue [27]. We found that the level of pre-rRNA synthesis increased with the overexpression of wildtype PCAF, while overexpression of the HAT-deficient mutant (Y616A/Y617A) and bromodomain mutant (V752A) did not affect pre-rRNA synthesis (Figure 4B). Significantly, co-expression of CSB with PCAF further augmented transcription (Figure 4C), suggesting that PCAF and CSB cooperated in Pol I transcription activation. These results demonstrated that both HAT and bromodomain of PCAF are required for activation of Pol I transcription, and that functional interplay of PCAF and CSB plays an important role in stimulating rDNA transcription.

Next, we examined whether the enzyme activity of PCAF was required for the association of Pol I with rDNA. The results showed that both PCAF mutants lost the ability to bind to rDNA promoter and coding region (Figure 4D, Figure S4C). Accordingly, overexpression of wildtype PCAF increased Pol I occupancy at rDNA repeats, but overexpression of mutants did not affect the association of Pol I with rDNA (Figure 4E, Figure S4D). Consistent with this, enrichment of Pol I was lower when PCAF was depleted (Figure 2C). Therefore, these results demonstrated that the HAT and bromodomain of PCAF are required for the recruitment of PCAF and Pol I to rDNA promoters.

### PCAF Stimulates rRNA Gene Transcription by Inducing Histone Acetylation

**Figure 5:**
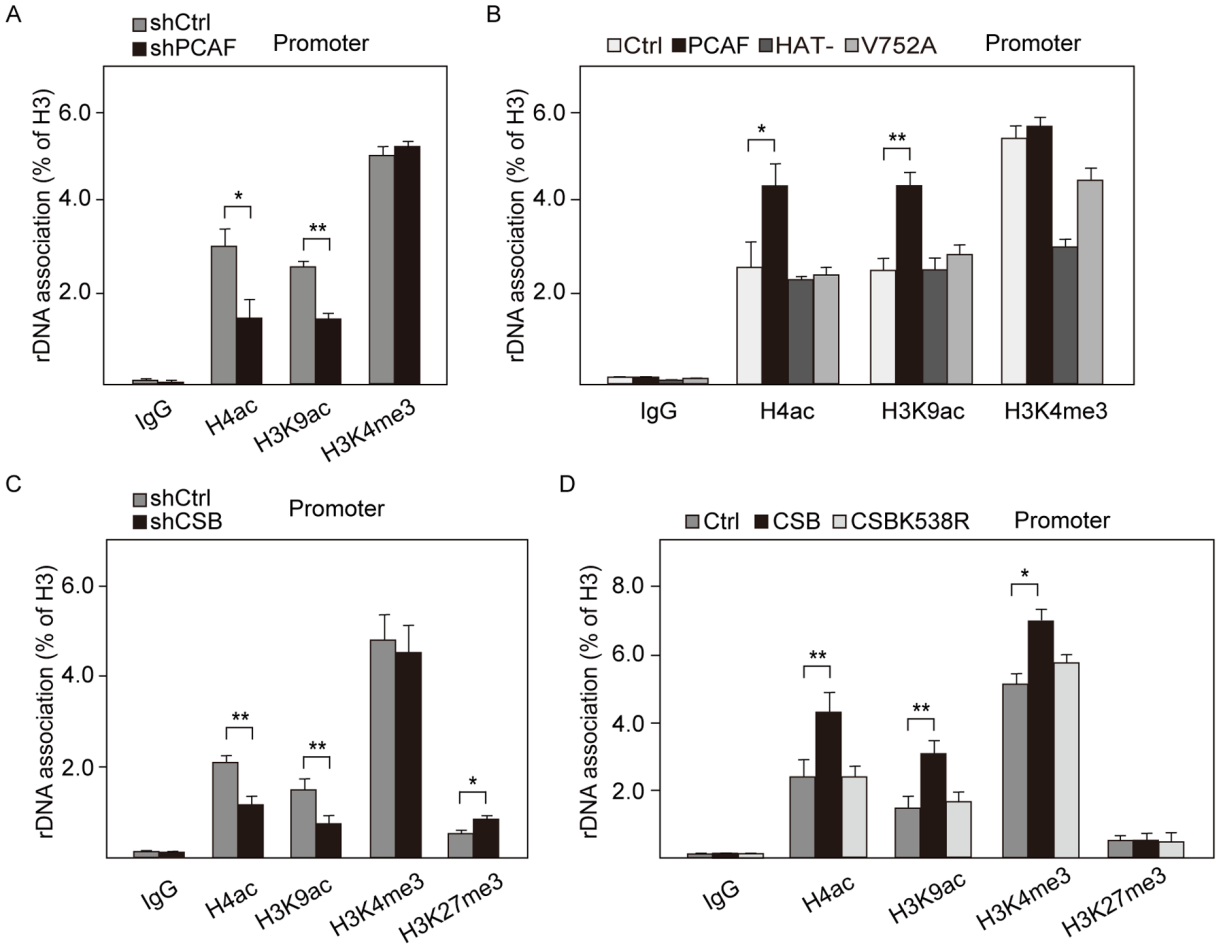
PCAF stimulates rRNA gene transcription by histone acetylation. A. Depletion of PCAF decreases the binding of H4ac and H3K9ac to the rDNA promoter. ChIP data show the associations of H4ac, H3K9ac and H3K4me3 with rDNA promoter in NIH 3T3 cells infected with lentiviruses encoding PCAF-specific shRNA (black bars) or control shRNA (gray bars). The immunoprecipitated DNA from PCAF knockdown cells (shPCAF) and control cells (shCtrl) was normalized to histone H3. Values of the average %IP (±standard deviation) for indicated histone modifications are shown with the standard deviation from three independent experiments. *P value <0.05, **P value <0.01. B. Overexpression of PCAF promotes the binding of H4ac and H3K9ac to the rDNA promoter. Data from ChIP experiments showing the rDNA occupancy of H4ac, H3K9ac and H3K4me3 in NIH 3T3 cells overexpressing wildtype PCAF and two mutants. The levels of histone modifications from PCAF overexpressed cells and mock-transfected cells (Ctrl) were normalized to histone H3. Error bars represent standard deviation (n = 3). *P value <0.05, **P value <0.01. C. Depletion of CSB reduces the levels of histone H4 acetylation and histone H3K9 acetylation at rDNA promoter. Cross-linked chromatins from NIH 3T3 cells after shRNA-mediated depletion of CSB (doxycycline-treated, 1 µg/ml, 72 hr) were immunoprecipitated with antibodies against H4ac, H3K9ac, H3K4me3 and H3K27me3. The immunoprecipitated DNA with specific histone modifications antibodies from CSB knockdown cells (shCSB) and control cells without treatment with tetracycline (shCtrl) was normalized to histone H3. The levels of indicated histone modifications are shown with the standard deviation from three independent experiments. *P value <0.05, **P value <0.01. D. Overexpression of CSB increases the associations of H4ac and H3K9ac with rDNA promoter. Cross-linked chromatins were from NIH 3T3 cells overexpressing wildtype or mutant CSB. Data from ChIP experiments show rDNA occupancy of indicated histone modifications in CSB overexpressed cells and mock-transfected cells (Ctrl). The levels of indicated histone modifications normalized to histone H3 are shown with the standard deviation from three independent experiments. *P value <0.05, **P value <0.01.

Histone acetylation plays an active role in rDNA transcription. PCAF is a histone acetyltransferase. Its association with active rRNA genes indicated that PCAF might be involved in maintaining H4 acetylation and/or H3K9 acetylation in active rDNA promoters. Indeed, knockdown of PCAF decreased the levels of H4 acetylation and H3K9 acetylation at rDNA promoter and coding region (Figure 5A, Figure S5A, Figure S5B). While overexpression of wildtype PCAF led to the increased levels of H4 acetylation and H3K9 acetylation, overexpression of HAT-deficient mutant (Y616A/Y617A) and bromodomain mutant (V752A) did not (Figure 5B, Figure S5C), demonstrating that PCAF was responsible for H4 acetylation and H3K9 acetylation at rDNA. Consistently, knockdown of CSB also decreased both H4 acetylation and H3K9 acetylation (Figure 5C, Figure S5D), while overexpression of CSB increased the levels of H4 acetylation and H3K9 acetylation (Figure 5D, Figure S5E). Given that CSB recruited PCAF to active rDNA promoters (Figure 2), these results demonstrated that CSB can recruit histone acetyltransferase PCAF to induce histone acetylation in active rDNA promoters.

## Discussion

Acetylation of histones plays a primary role in nucleosomal conformation changes, which enhance the accessibility of transcription factors to their recognition sites [23], [28]. The poised state of rRNA genes is mediated by the nucleosomal remodeling and deacetylation (NuRD) complex. Such poised promoters are unmethylated, associated with components of the pre-initiation complex, marked by bivalent histone modifications such as H3K4me3 and H3K27me3, but lack histone acetylation. The active state of rRNA genes, however, is associated with components of Pol initiation complex, marked by unmethylated DNA and euchromatic features associated with histone acetylation [5]. It is obvious that there is a correlation between histone acetylation and the loading of Pol I to active promoters when poised promoters are activated.

**Figure 6:**
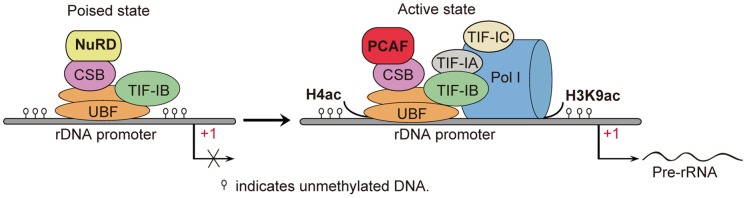
Schematic models for the role of PCAF and CSB in rRNA transcription. Deacetylation of H4 and H3K9 are associated with poised rDNA promoters, whereas acetylation of H4 and H3K9 are restricted to rDNA promoters in active state. After conversion of poised rDNA promoters to active ones, PCAF is recruited to active state of rRNA promoters by CSB, and induces acetylation of histones to promote association of RNA polymerase I with rDNA promoters, thereby facilitating the initiation of rDNA transcription.

In this study, we found that hyperacetylation of histones H4 and H3K9 in active rDNA promoters was induced by histone acetyltransferase PCAF. In addition, CSB interacted with PCAF, and knockdown of CSB reduced the binding of PCAF to rDNA promoters. Overexpression of CSB increased the level of PCAF binding to rDNA, indicating that PCAF was recruited to rDNA by CSB. Furthermore, CSB mediated the interaction between PCAF and Pol I. Knockdown of either CSB or PCAF impaired the association of Pol I with rDNA and inhibited pre-rRNA synthesis. Moreover, PCAF was required for acetylation of H4 and H3K9, and these modifications were required for the loading of Pol I to rDNA promoters and activation of Pol I transcription. Finally, sequential ChIP revealed that CSB and PCAF occupied the same region on active rDNA promoter sequences. Based on these data, we propose that the transition from poised rDNA promoters to active ones is accompanied with the recruitment of PCAF by CSB, resulting in acetylation of histones and assembly of Pol I transcription initiation complex (Figure 6). Our results demonstrated that CSB and PCAF participate in the conversion of rRNA genes from poise to active state by histone acetylation.

Previous studies have demonstrated that the transcription factor TTF-I recognizes the rDNA terminator sequence known as T_0_ [1], [4]. Binding of TTF-I to the T_0_ element results in the recruitment of ATP-dependent nucleosome remodeling complexes to promoters. TTF-I recruits chromatin modifier CSB, bivalent chromatin maintainer CHD4 or the repressive remodeling complex NoRC to establish characteristic chromatin structure at active, poised or silent rRNA genes, respectively [5], [15], [29]. Given that CSB targets PCAF to rDNA, and CSB is recruited to rDNA by TTF-I [15], the association of PCAF with rDNA may fundamentally require both TTF1 and CSB. Indeed, it has been shown that TTF1 interacts with PCAF both *in vivo* and *in vitro*, and targets PCAF to the rDNA promoter [30]. More interestingly, PCAF is able to acetylate TAFI68 *in vitro*, the second largest subunit of the TATA box-binding protein (TBP)-containing factor TIF-IB/SL1, and this acetylation enhances the binding of TAFI68 to the rDNA promoter and promotes Pol I transcription [30]. However, overexpression of PCAF stimulates transcription *in vivo*, but recombinant PCAF does not stimulate rDNA transcription *in vitro* in the absence of nucleosomes [22]. Moreover, acetylated TAFI68 by PCAF strongly binds to DNA fragment covering murine rDNA sequences regardless of the presence or absence of nucleosomes [30]. These data suggest that PCAF affects rDNA transcription at least partially depending on the chromatin. More likely PCAF regulates rDNA transcription by affecting acetylation of both core histones of ribosomal chromatin as well as chromatin associated non-histone proteins such as TAFI68 in a chromatin-dependent or -independent manner.

Chromatin remodeling factors are required for the opening of chromatin structure in rDNA promoters. These factors affect transcription by recruitment of enzymes that modify histone methylation and acetylation [5], [15], [29]. For example, TIP5, the large subunit of the nucleolar remodeling complex NoRC, mediates histone deacetylation at rDNA locus to establish a repressed chromatin structure [29]. The chromatin remodeling complex B-WICH, which comprises the William syndrome transcription factor (WSTF) and SNF2h, allows H3 acetylation of rRNA genes to activate rRNA gene transcription [23]. Further, the associations of histone acetyl-transferases PCAF, p300 and GCN5 with the promoter are reduced in WSTF knockdown cells, but WSTF does not interact with PCAF and GCN5, suggesting that B-WICH is not a direct recruiter of HATs [23]. In this study, we found that CSB interacted directly with PCAF. Knockdown of CSB decreased the association of PCAF with rDNA promoter and coding region, demonstrating that CSB is the recruiter of PCAF, which leads to acetylation of H4 and H3K9 at rDNA. Notably, CSB plays a dual role in RNA Pol I transcription. At promoters, CSB cooperates with CHD4/NuRD to establish the poised state of rRNA gene promoters characterized by bivalent histone modifications and altered nucleosomal positions [5]. In coding regions, CSB is associated with active rRNA genes and recruits the methyltransferase G9a, which contributes to the elongation of RNA Pol I transcription [15]. Consistently, we found that CSB not only targeted PCAF to promoters, but also to coding regions. Knockdown of PCAF decreases the levels of histone acetylation, and impairs the association of Pol I with coding regions. Thus, our data tend to predict that PCAF functions on coding regions in a manner similar to the promoters. However, the molecular mechanism underlying the manner of cooperation between PCAF with CSB and G9a for transcriptional elongation remains to be further studied.

Bromodomain is a protein domain that recognizes acetylated lysine residues [27], [31]. Such recognition of lysine acetylation is often a prerequisite for protein-histone association and chromatin remodeling. Bromodomain is also present in PCAF. Our results showed that defective bromodomain as well as HAT activity disrupted the targeting of PCAF to rDNA promoters, demonstrating that recruitment of PCAF to promoters requires the binding of bromodomain to acetylated histone tails mediated by HAT domain activity. Bromodomain induces full binding and remodeling activity of Swi2/Snf2 complex on H3- and H4-acetylated nucleosomes [32]. As a member of SWI2/SNF2 family, CSB requires lysine acetylation to bind to active nucleosomes of rDNA promoters. Indeed, our results showed that overexpression of PCAF induced higher binding of CSB to rDNA promoters, but CSB is able to bind to both non-acetylated and acetylated promoters [5], suggesting that CSB might use alternate ways to bind to non-acetylated histone nucleosomes, which counteract PCAF acetylation pathway. Further studies are needed to prove the postulated dynamics of nucleosomal architecture mediated by histone modifications and chromatin remodelers.

## Materials and Methods

### Plasmids and Cell Lines

NIH 3T3 cells in which CSB is knocked down by doxycycline treatment were generated by transfection with pcDNA4-TR and an expression vector encoding shRNA against CSB. The expression vector pCX-Flag-PCAF was provided by Prof. Grummt (German Cancer Research Center). The cDNA encoding CSB, CSBK538R, PCAF, HAT domain deficient mutant (Y616A/Y617A) and bromodomain mutant (V752A) were subcloned into the pBABE-Puro retroviral vector.

### Knockdown of PCAF by shRNA

Lentiviruses were generated with 293T cells by transfection with vectors encoding PCAF shRNA or control shRNA. NIH 3T3 cells were infected with lentiviruses and selected by puromycin.

### Northern Blot Hybridization

Non-isotopic labeling of the DNA probes with digoxigenin (DIG) was achieved by using DIG-11-dUTP (Roche Diagnostics, USA). Specific probes for 45S pre-rRNA were obtained by amplifying the sequences from pMr600 provided by Prof. Grummt (German Cancer Research Center). Total RNA was electrophoresed on a 1.2% formaldehyde agarose gel, capillary-blotted onto a Hybond membrane (Amersham) and UV cross-linked. Blots were hybridized with 45S pre-rRNA probes. The signal was developed with DIG High Prime DNA Labeling and Detection Starter Kit I (Roche Diagnostics, USA). Primers used for Northern blot hybridization are shown in Table S1.

### Transfections and RNA Analysis

For overexpression of proteins, retroviruses were generated with Plat-E packaging cells by transfection with vectors encoding wildtype CSB, CSB ATPase-deficient mutant (CSBK538R), wildtype PCAF, PCAF HAT domain mutant (Y616A/Y617A) and bromodomain mutant (V752A). NIH 3T3 cells were infected with retroviruses and harvested after 8 days. After washing twice with ice-cold PBS, cells were lysed in 1 ml TRIzol reagent (Invitrogen) followed by RNA extraction. 45S pre-rRNA and GAPDH mRNA were measured by qRT-PCR. Primers are shown in Table S1.

### Immunoprecipitation

Nuclear extracts from 293T cells were pre-cleared by incubation with protein A/G Sepharose at 4°C for 1 hr and incubated overnight with respective antibodies at 4°C. Protein A/G Sepharose was then added to the lysates and incubated with agitation for 4 hr at 4°C. After washing with 200 mM NaCl lysis buffer (20 mM Tris-HCl [pH 8.0], 200 mM NaCl, 2 mM EDTA, 2 mM EGTA, 1% Triton X-100 and complete protease inhibitor cocktail), bound proteins were eluted from beads by boiling in SDS-PAGE loading buffer and analyzed on western blots.

### Immunofluorescence

U2OS cells grown on coverslips were rinsed briefly in PBS and fixed with acetone for 10 min at −20°C. After washing three times with PBS, cells were blocked in 1% BSA for 1 hr at room temperature and incubated with primary antibodies overnight at 4°C. The cells were then washed three times with PBST (1×PBS, 0.1%Triton X-100) and incubated with secondary antibodies for 1 hr at room temperature in dark. After washing three times with PBST, the cells were counterstained with 1 µg/ml DAPI for 3 min. The coverslips were rinsed in PBS and mounted onto slides with mounting medium (Southern Biotech). PCAF, CSB, and Pol I were visualized by indirect immunofluorescence.

### Chromatin Immunoprecipitation (ChIP)

Chromatins cross-linked by 1.5% formaldehyde were sonicated or digested with micrococcal nuclease (Roche). We used 50 µg chromatins for each ChIP and saved 20% of the chromatins as input. After pre-cleared, the chromatins were incubated overnight with antibodies at 4°C followed by a second incubation with protein A/G Sepharose for 1 hr. Protein-DNA complex was washed on a rotating platform at 4°C for 8 min consecutively with 1 ml of each of the following solutions: low salt wash buffer (150 mM NaCl, 20 mM Tris-HCl [pH 8.0], 2 mM EDTA, 1% Triton X-100 and 0.1% SDS), high salt wash buffer (500 mM NaCl, 20 mM Tris-HCl [pH 8.0], 2 mM EDTA, 1% Triton X-100 and 0.1% SDS), LiCl wash buffer (250 mM LiCl, 10 mM Tris-HCl [pH 8.0], 1 mM EDTA, 1% Na-deoxycholate and 1% NP-40) and twice with TE (10 mM Tris-HCl [pH 8.0], 1 mM EDTA). Chelex 100 slurry (Biorad) was used to reverse the cross-linked chromatin. Bead-bound DNA was boiled for 10 min in 100 µl of 10% Chelex slurry. Proteins were then removed by digestion with Proteinase K at 55°C for 30 min followed by boiling for 10 min to inactivate Proteinase K. About 70 µl of the supernatant was transferred to a fresh tube. DNA was then amplified by qRT- PCR using primers used for ChIP. Primers and their sequences are shown in Table S1.

### Methylation-sensitive ChIP-Chop

ChIP DNA samples (8 µl each) were either mock-treated or digested with 20 units of *Hpa* II (New England Lab) overnight at 37°C. Amplification was performed using primers that amplified mouse rDNA from −165/+16 (the transcription start site was set as +1), a region covering one *Hpa* II restriction site (analyzed fragment primers). For normalization, a DNA fragment harboring rDNA sequence from −105 to +16 containing no *Hpa* II restriction site was also amplified (control fragment primers). Percentage *Hpa* II resistance was calculated as follows: *Hpa* II = [(A*Hpa* II/Amock)/(C*Hpa* II/Cmock)] ×100. A: analyzed fragment primers, C: control fragment primers. Primers used are shown in Table S1.

### *Hpa* II Digestion

PCAF or CSB was overexpressed in NIH 3T3 cells infected with retrovirus. Genomic DNA was extracted and digested with 20 units of *Hpa* II (New England Lab) overnight at 37°C. Amplification was performed using primers that amplified mouse rDNA promoter or coding region. The relative resistance to *Hpa* II digestion was calculated after normalization to mock-digested DNA.

### Statistical Method

Statistical analyses were performed using the Graphpad Prism 5 software package to analyze the significance of data.

### Antibodies

Antibodies against Pol I (SC-28714, SC-48385), UBF (SC-9131X), PCAF (SC-8999X, SC-13124), CSB (SC-25370), GCN5 (SC-20698X) and p300 (SC-585) were from Santa Cruz. Anti-H3, anti-H3K4me3 and anti-H3K27me3 were from Abcam. Antibodies against H4ac and H3K9ac were from Upstate Biotechnology.

## Supporting Information

Figure S1 **PCAF interacts with CSB and Pol I in mouse NIH 3T3 cells.** Nuclear extracts from NIH 3T3 cells were incubated with control IgGs, or anti-PCAF antibodies. About 10% of input and 80% of precipitated proteins were analyzed on western blots.
(TIF)

Figure S2 **CSB recruits PCAF to rDNA coding region.** A. Inducible knockdown of CSB impairs Pol I and PCAF occupancy at rDNA coding region. ChIP experiment was from NIH 3T3 cells treated with tetracycline to induce CSB knockdown (doxycycline-treated, 1 µg/ml, 72 hr). qRT-PCR data show the associations of CSB, UBF, Pol I, PCAF, GCN5 and p300 with rDNA coding region. The levels of indicated proteins from CSB knockdown cells (shCSB) and control cells without treatment with tetracycline (shCtrl) were normalized to input DNA. Error bars represent standard deviation (n = 3). *P value <0.05, **P value <0.01. B. Overexpression of CSB increases the associations of Pol I and PCAF with rDNA coding region. ChIP data were from NIH 3T3 cells overexpressing wildtype or mutant CSB. Values of the average %IP (±standard deviation) for indicated proteins from CSB overexpressed cells and mock-transfected cells (Ctrl) were normalized to input DNA. Error bars represent standard deviation (n = 3). *P value <0.05, **P value <0.01. C. Knockdown of PCAF decreases the binding of Pol I, but not UBF and CSB to the rDNA coding region. ChIP assay showing the associations of UBF, Pol I, PCAF and CSB with rDNA coding region after knockdown of PCAF using PCAF-specific shRNA in NIH 3T3 cells. The levels of indicated proteins from PCAF knockdown cells (shPCAF) and control cells (shCtrl) were normalized to input DNA. Error bars represent standard deviation (n = 3). *P value <0.05, **P value <0.01. D. Depletion of PCAF decreases the binding of Pol I, but not UBF and CSB to the rDNA in 293T cells. ChIP data were from 293T cells after siRNA-mediated depletion of PCAF. The levels of indicated proteins from PCAF knockdown cells (siPCAF) and control cells (siCtrl) were normalized to input DNA. Error bars represent standard deviation (n = 3). *P value <0.05, **P value <0.01. E. Association of CSB with Pol I does not depends on PCAF. Nuclear extracts from NIH 3T3 cells infected with either control shRNA or PCAF-specific shRNA were incubated with anti-CSB antibodies or control IgGs. About 10% of input and 80% of precipitated proteins were analyzed on western blots.
(TIF)

Figure S3 **PCAF and CSB tend to bind to unmethylated rDNA at coding region.** Cross-linked chromatin from NIH 3T3 cells was precipitated with the indicated antibodies and mock digested or digested with *Hpa* II before PCR amplification. Relative levels of *Hpa* II-resistant, inactive rDNA copies (black bars) and unmethylated, active copies (gray bars) were determined by qRT-PCR using primer pairs flanking the *Hpa II* sites on rDNA coding region. Error bars represent standard deviation (n = 3).
(TIF)

Figure S4 **The association of PCAF with rDNA coding region requires HAT and bromodomain. A.** Depletion of CSB or PCAF impairs 45S pre-rRNA synthesis. CSB was reduced in NIH 3T3 cells by tetracycline-induced synthesis of CSB-specific shRNA (shCSB). Cells were treated with 1 µg/ml doxycycline (dox) for 48 hr (left). On the right, 293T cells were transfected with PCAF-specific siRNAs (siPCAF #1 and #2) or control siRNA (siCtrl). 45S pre-rRNA synthesis was measured by qRT-PCR and normalized to GAPDH mRNA (n = 3). **P value <0.01, ***P value <0.001. Knockdown of CSB or PCAF was determined by western blot analysis using antibodies against Pol I, UBF, and tubulin. B. Knockdown of CSB or PCAF impairs nucleolar transcription. NIH 3T3 cells with knockdown of CSB (shCSB) or PCAF (shPCAF) were labeled for 15 min with fluorouridine (FUrd), and stained with antibodies to BrdU. Bar diagram represents intensity of FUrd labeling signals. Scale bar, 5 µm. **P value <0.01. C. PCAF mutants do not bind to rDNA coding region. ChIP data show the occupancy of PCAF and PCAF mutants on rDNA coding region in NIH 3T3 cells overexpressing wildtype PCAF, HAT-deficient mutant or bromodomain mutant. The immunoprecipitated DNA from PCAF overexpressed cells and mock-transfected cells (Ctrl) was normalized to input DNA. The levels of indicated proteins are shown with the standard deviation from three independent experiments. *P value <0.05. D. Overexpression of PCAF increases the occupancy of Pol I and CSB on rDNA coding region. ChIP data show the occupancy of UBF, Pol I and CSB on rDNA coding region in NIH 3T3 cells overexpressing wildtype PCAF, HAT-deficient mutant or bromodomain mutant. The levels of indicated proteins from PCAF overexpressed cells and mock-transfected cells (Ctrl) were normalized to input DNA (n = 3). *P value <0.05.
(TIF)

Figure S5 **PCAF stimulates rRNA gene transcription by correlating with histone acetylation at the coding region.** A. Depletion of PCAF decreases the binding of H4ac and H3K9ac to the rDNA coding region. ChIP data show the associations of H4ac, H3K9ac and H3K4me3 with rDNA coding region in NIH 3T3 cells infected with lentiviruses encoding PCAF-specific shRNA (black bars) or control shRNA (gray bars). Values of the average %IP (±standard deviation) for histone modifications from PCAF knockdown cells (shPCAF) and control cells (shCtrl) were normalized to histone H3. Error bars represent standard deviation (n = 3). *P value <0.05, **P value <0.01. B. Knockdown of PCAF decreases the binding of H4ac and H3K9ac to the rDNA. ChIP data were from 293T cells after siRNA-mediated depletion of PCAF. The levels of histone modifications from PCAF knockdown cells (siPCAF) and control cells (siCtrl) were normalized to histone H3. Error bars represent standard deviation (n = 3). *P value <0.05, **P value <0.01. C. Overexpression of PCAF promotes the binding of H4ac and H3K9ac to the rDNA coding region. Data from ChIP experiments show the rDNA occupancy of H4ac, H3K9ac and H3K4me3 in NIH 3T3 cells overexpressing wildtype PCAF and two mutants. The levels of the average %IP (±standard deviation) for H4ac, H3K9ac and H3K4me3 from PCAF overexpressed cells and mock-transfected cells (Ctrl) were normalized to histone H3. Error bars represent standard deviation (n = 3). *P value <0.05, **P value <0.01. D. Depletion of CSB reduces the levels of histone H4 acetylation and histone H3K9 acetylation at rDNA coding region. Cross-linked chromatins from NIH 3T3 cells after shRNA-mediated depletion of CSB were immunoprecipitated with antibodies against H4ac, H3K9ac, H3K4me3 and H3K27me3. The levels of histone modifications from CSB knockdown cells (shCSB) and control cells (shCtrl) were normalized to histone H3. Error bars represent standard deviation (n = 3). **P value <0.01. E. Overexpression of CSB increases the associations of H4ac and H3K9ac with rDNA coding region. Cross-linked chromatins were from NIH 3T3 cells overexpressing wildtype or mutant CSB. ChIP data for the different histone modifications from CSB overexpressed cells and mock-transfected cells (Ctrl) were normalized to histone H3. The levels of indicated histone modifications are shown with the standard deviation from three independent experiments. **P value <0.01.
(TIF)

Table S1 Primer lists.
(DOCX)

Table S2 Values of the average % IP (± standard deviation) normalized to input DNA for three independent experiments are shown.
(DOCX)

Methods and References S1 (DOC)

## References

[1] Grummt I (2003) Life on a planet of its own: regulation of RNA polymerase I transcription in the nucleolus. Genes Dev 17: 1691–1702. 12865296 10.1101/gad.1098503R

[2] Moss T (2004) At the crossroads of growth control; making ribosomal RNA. Curr Opin Genet Dev 14: 210–217. 15196469 10.1016/j.gde.2004.02.005

[3] Russell J, Zomerdijk JC (2006) The RNA polymerase I transcription machinery. Biochem Soc Symp: 203–216. 10.1042/bss0730203 PMC3858827 16626300

[4] McStay B, Grummt I (2008) The epigenetics of rRNA genes: from molecular to chromosome biology. Annu Rev Cell Dev Biol 24: 131–157. 18616426 10.1146/annurev.cellbio.24.110707.175259

[5] Xie W, Ling T, Zhou Y, Feng W, Zhu Q, et al (2012) The chromatin remodeling complex NuRD establishes the poised state of rRNA genes characterized by bivalent histone modifications and altered nucleosome positions. Proc Natl Acad Sci U S A 109: 8161–8166. 22570494 10.1073/pnas.1201262109 PMC3361413

[6] Pazin MJ, Kadonaga JT (1997) SWI2/SNF2 and related proteins: ATP-driven motors that disrupt protein-DNA interactions? Cell 88: 737–740. 9118215 10.1016/s0092-8674(00)81918-2

[7] Citterio E, Van Den Boom V, Schnitzler G, Kanaar R, Bonte E, et al (2000) ATP-dependent chromatin remodeling by the Cockayne syndrome B DNA repair-transcription-coupling factor. Mol Cell Biol 20: 7643–7653. 11003660 10.1128/mcb.20.20.7643-7653.2000 PMC86329

[8] Newman JC, Bailey AD, Weiner AM (2006) Cockayne syndrome group B protein (CSB) plays a general role in chromatin maintenance and remodeling. Proc Natl Acad Sci U S A 103: 9613–9618. 16772382 10.1073/pnas.0510909103 PMC1480455

[9] Venema J, Mullenders LH, Natarajan AT, van Zeeland AA, Mayne LV (1990) The genetic defect in Cockayne syndrome is associated with a defect in repair of UV-induced DNA damage in transcriptionally active DNA. Proc Natl Acad Sci U S A 87: 4707–4711. 2352945 10.1073/pnas.87.12.4707 PMC54186

[10] Laine JP, Egly JM (2006) When transcription and repair meet: a complex system. Trends Genet 22: 430–436. 16797777 10.1016/j.tig.2006.06.006

[11] Yu A, Fan HY, Liao D, Bailey AD, Weiner AM (2000) Activation of p53 or loss of the Cockayne syndrome group B repair protein causes metaphase fragility of human U1, U2, and 5S genes. Mol Cell 5: 801–810. 10882116 10.1016/s1097-2765(00)80320-2

[12] Tantin D, Kansal A, Carey M (1997) Recruitment of the putative transcription-repair coupling factor CSB/ERCC6 to RNA polymerase II elongation complexes. Mol Cell Biol 17: 6803–6814. 9372911 10.1128/mcb.17.12.6803 PMC232536

[13] Selby CP, Sancar A (1997) Cockayne syndrome group B protein enhances elongation by RNA polymerase II. Proc Natl Acad Sci U S A 94: 11205–11209. 9326587 10.1073/pnas.94.21.11205 PMC23417

[14] Bradsher J, Auriol J, Proietti de Santis L, Iben S, Vonesch JL, et al (2002) CSB is a component of RNA pol I transcription. Mol Cell 10: 819–829. 12419226 10.1016/s1097-2765(02)00678-0

[15] Yuan X, Feng W, Imhof A, Grummt I, Zhou Y (2007) Activation of RNA polymerase I transcription by cockayne syndrome group B protein and histone methyltransferase G9a. Mol Cell 27: 585–595. 17707230 10.1016/j.molcel.2007.06.021

[16] Shahbazian MD, Grunstein M (2007) Functions of site-specific histone acetylation and deacetylation. Annu Rev Biochem 76: 75–100. 17362198 10.1146/annurev.biochem.76.052705.162114

[17] Roth SY, Denu JM, Allis CD (2001) Histone acetyltransferases. Annu Rev Biochem 70: 81–120. 11395403 10.1146/annurev.biochem.70.1.81

[18] Carrozza MJ, Utley RT, Workman JL, Cote J (2003) The diverse functions of histone acetyltransferase complexes. Trends Genet 19: 321–329. 12801725 10.1016/S0168-9525(03)00115-X

[19] Wang Z, Zang C, Cui K, Schones DE, Barski A, et al (2009) Genome-wide mapping of HATs and HDACs reveals distinct functions in active and inactive genes. Cell 138: 1019–1031. 19698979 10.1016/j.cell.2009.06.049 PMC2750862

[20] Ianari A, Gallo R, Palma M, Alesse E, Gulino A (2004) Specific role for p300/CREB-binding protein-associated factor activity in E2F1 stabilization in response to DNA damage. J Biol Chem 279: 30830–30835. 15123636 10.1074/jbc.M402403200

[21] Liu L, Scolnick DM, Trievel RC, Zhang HB, Marmorstein R, et al (1999) p53 sites acetylated in vitro by PCAF and p300 are acetylated in vivo in response to DNA damage. Mol Cell Biol 19: 1202–1209. 9891054 10.1128/mcb.19.2.1202 PMC116049

[22] Hirschler-Laszkiewicz I, Cavanaugh A, Hu Q, Catania J, Avantaggiati ML, et al (2001) The role of acetylation in rDNA transcription. Nucleic Acids Res 29: 4114–4124. 11600700 10.1093/nar/29.20.4114 PMC60214

[23] Vintermist A, Bohm S, Sadeghifar F, Louvet E, Mansen A, et al (2011) The chromatin remodelling complex B-WICH changes the chromatin structure and recruits histone acetyl-transferases to active rRNA genes. PLoS One 6: e19184. 21559432 10.1371/journal.pone.0019184 PMC3084792

[24] Chen D, Belmont AS, Huang S (2004) Upstream binding factor association induces large-scale chromatin decondensation. Proc Natl Acad Sci U S A 101: 15106–15111. 15477594 10.1073/pnas.0404767101 PMC524054

[25] Santoro R, Grummt I (2001) Molecular mechanisms mediating methylation-dependent silencing of ribosomal gene transcription. Mol Cell 8: 719–725. 11583633 10.1016/s1097-2765(01)00317-3

[26] Korzus E, Torchia J, Rose DW, Xu L, Kurokawa R, et al (1998) Transcription factor-specific requirements for coactivators and their acetyltransferase functions. Science 279: 703–707. 9445475 10.1126/science.279.5351.703

[27] Mujtaba S, He Y, Zeng L, Farooq A, Carlson JE, et al (2002) Structural basis of lysine-acetylated HIV-1 Tat recognition by PCAF bromodomain. Mol Cell 9: 575–586. 11931765 10.1016/s1097-2765(02)00483-5

[28] Vettese-Dadey M, Grant PA, Hebbes TR, Crane- Robinson C, Allis CD, et al (1996) Acetylation of histone H4 plays a primary role in enhancing transcription factor binding to nucleosomal DNA in vitro. EMBO J 15: 2508–2518. 8665858 PMC450183

[29] Zhou Y, Santoro R, Grummt I (2002) The chromatin remodeling complex NoRC targets HDAC1 to the ribosomal gene promoter and represses RNA polymerase I transcription. EMBO J 21: 4632–4640. 12198165 10.1093/emboj/cdf460 PMC126197

[30] Muth V, Nadaud S, Grummt I, Voit R (2001) Acetylation of TAF(I)68, a subunit of TIF-IB/SL1, activates RNA polymerase I transcription. EMBO J 20: 1353–1362. 11250901 10.1093/emboj/20.6.1353 PMC145524

[31] Dey A, Chitsaz F, Abbasi A, Misteli T, Ozato K (2003) The double bromodomain protein Brd4 binds to acetylated chromatin during interphase and mitosis. Proc Natl Acad Sci U S A 100: 8758–8763. 12840145 10.1073/pnas.1433065100 PMC166386

[32] Awad S, Hassan AH (2008) The Swi2/Snf2 bromodomain is important for the full binding and remodeling activity of the SWI/SNF complex on H3- and H4-acetylated nucleosomes. Ann N Y Acad Sci 1138: 366–375. 18837912 10.1196/annals.1414.038

