# Supplementary figures and images for: The Chromatin Remodeling Factor CSB Recruits Histone Acetyltransferase PCAF to rRNA Gene Promoters in Active State for Transcription Initiation
Source: PLoS One. 2013 May 7;8(5):e62668. doi: 10.1371/journal.pone.0062668 (PMC3646882; doi:10.1371/journal.pone.0062668)

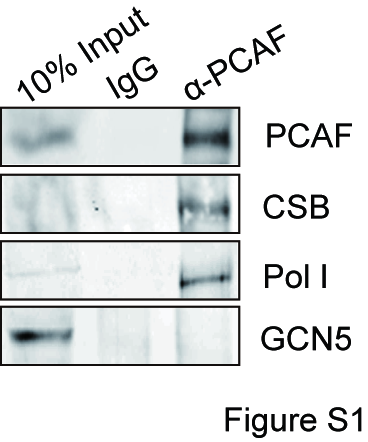

Supplement: Figure S1 — PCAF interacts with CSB and Pol I in mouse NIH 3T3 cells. Nuclear extracts from NIH 3T3 cells were incubated with control IgGs, or anti-PCAF antibodies. About 10% of input and 80% of precipitated proteins were analyzed on western blots. (TIF) [file pone.0062668.s001.tif]

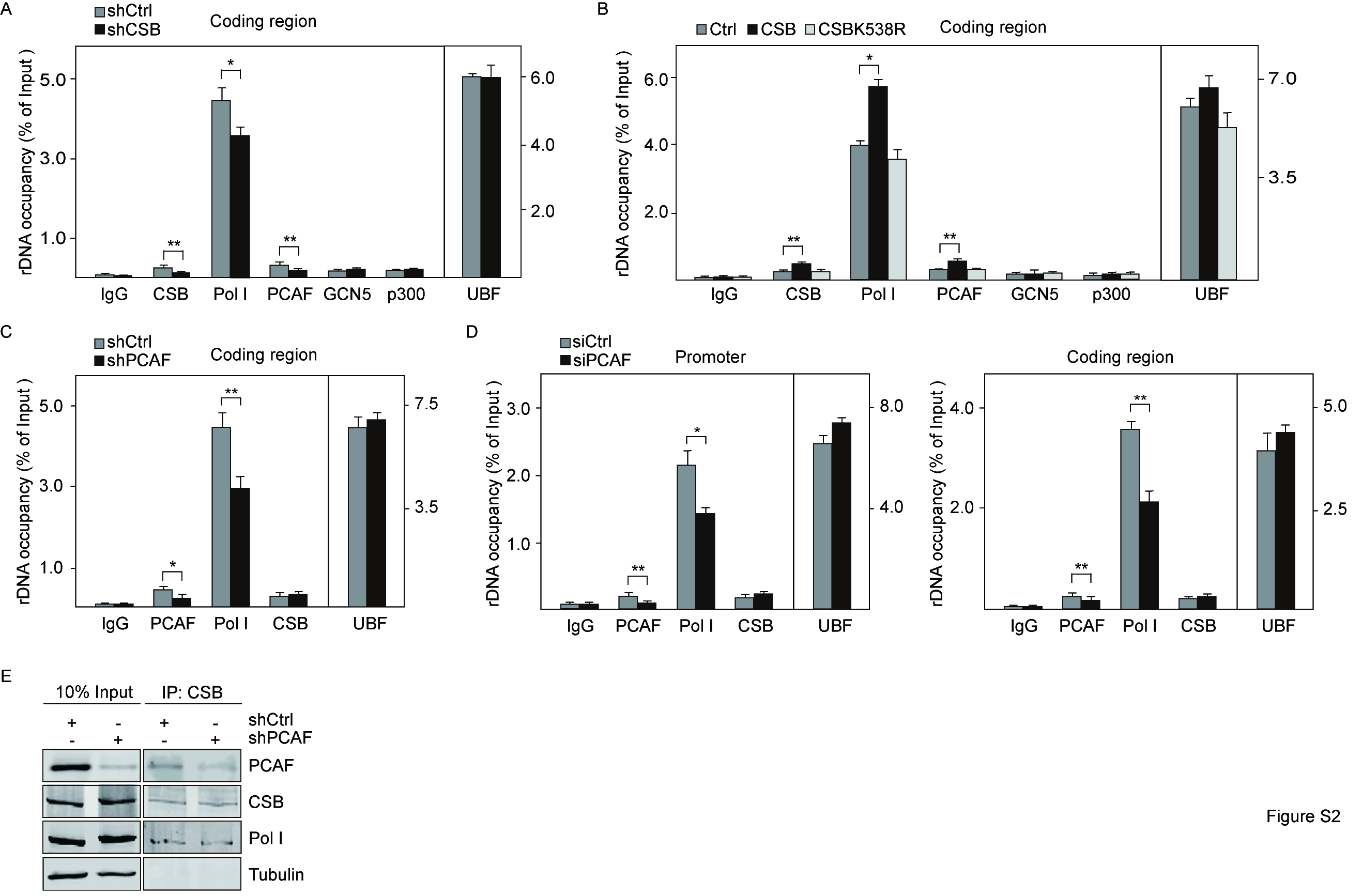

Supplement: Figure S2 — CSB recruits PCAF to rDNA coding region. A. Inducible knockdown of CSB impairs Pol I and PCAF occupancy at rDNA coding region. ChIP experiment was from NIH 3T3 cells treated with tetracycline to induce CSB knockdown (doxycycline-treated, 1 µg/ml, 72 hr). qRT-PCR data show the associations of CSB, UBF, Pol I, PCAF, GCN5 and p300 with rDNA coding region. The levels of indicated proteins from CSB knockdown cells (shCSB) and control cells without treatment with tetracycline (shCtrl) were normalized to input DNA. Error bars represent standard deviation (n = 3). *P value <0.05, **P value <0.01. B. Overexpression of CSB increases the associations of Pol I and PCAF with rDNA coding region. ChIP data were from NIH 3T3 cells overexpressing wildtype or mutant CSB. Values of the average %IP (±standard deviation) for indicated proteins from CSB overexpressed cells and mock-transfected cells (Ctrl) were normalized to input DNA. Error bars represent standard deviation (n = 3). *P value <0.05, **P value <0.01. C. Knockdown of PCAF decreases the binding of Pol I, but not UBF and CSB to the rDNA coding region. ChIP assay showing the associations of UBF, Pol I, PCAF and CSB with rDNA coding region after knockdown of PCAF using PCAF-specific shRNA in NIH 3T3 cells. The levels of indicated proteins from PCAF knockdown cells (shPCAF) and control cells (shCtrl) were normalized to input DNA. Error bars represent standard deviation (n = 3). *P value <0.05, **P value <0.01. D. Depletion of PCAF decreases the binding of Pol I, but not UBF and CSB to the rDNA in 293T cells. ChIP data were from 293T cells after siRNA-mediated depletion of PCAF. The levels of indicated proteins from PCAF knockdown cells (siPCAF) and control cells (siCtrl) were normalized to input DNA. Error bars represent standard deviation (n = 3). *P value <0.05, **P value <0.01. E. Association of CSB with Pol I does not depends on PCAF. Nuclear extracts from NIH 3T3 cells infected with either control shRNA or PCAF-specific sh [file pone.0062668.s002.tif]

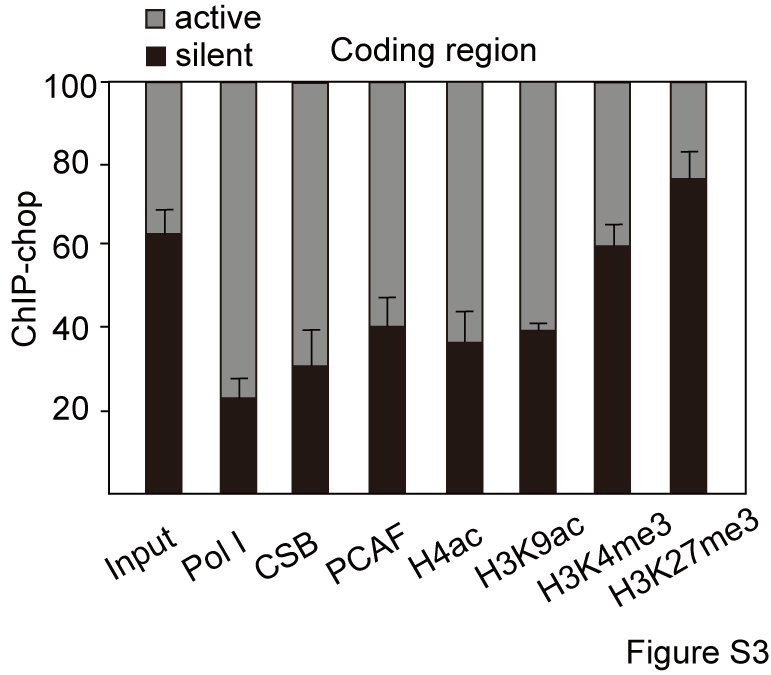

Supplement: Figure S3 — PCAF and CSB tend to bind to unmethylated rDNA at coding region. Cross-linked chromatin from NIH 3T3 cells was precipitated with the indicated antibodies and mock digested or digested with Hpa II before PCR amplification. Relative levels of Hpa II-resistant, inactive rDNA copies (black bars) and unmethylated, active copies (gray bars) were determined by qRT-PCR using primer pairs flanking the Hpa II sites on rDNA coding region. Error bars represent standard deviation (n = 3). (TIF) [file pone.0062668.s003.tif]

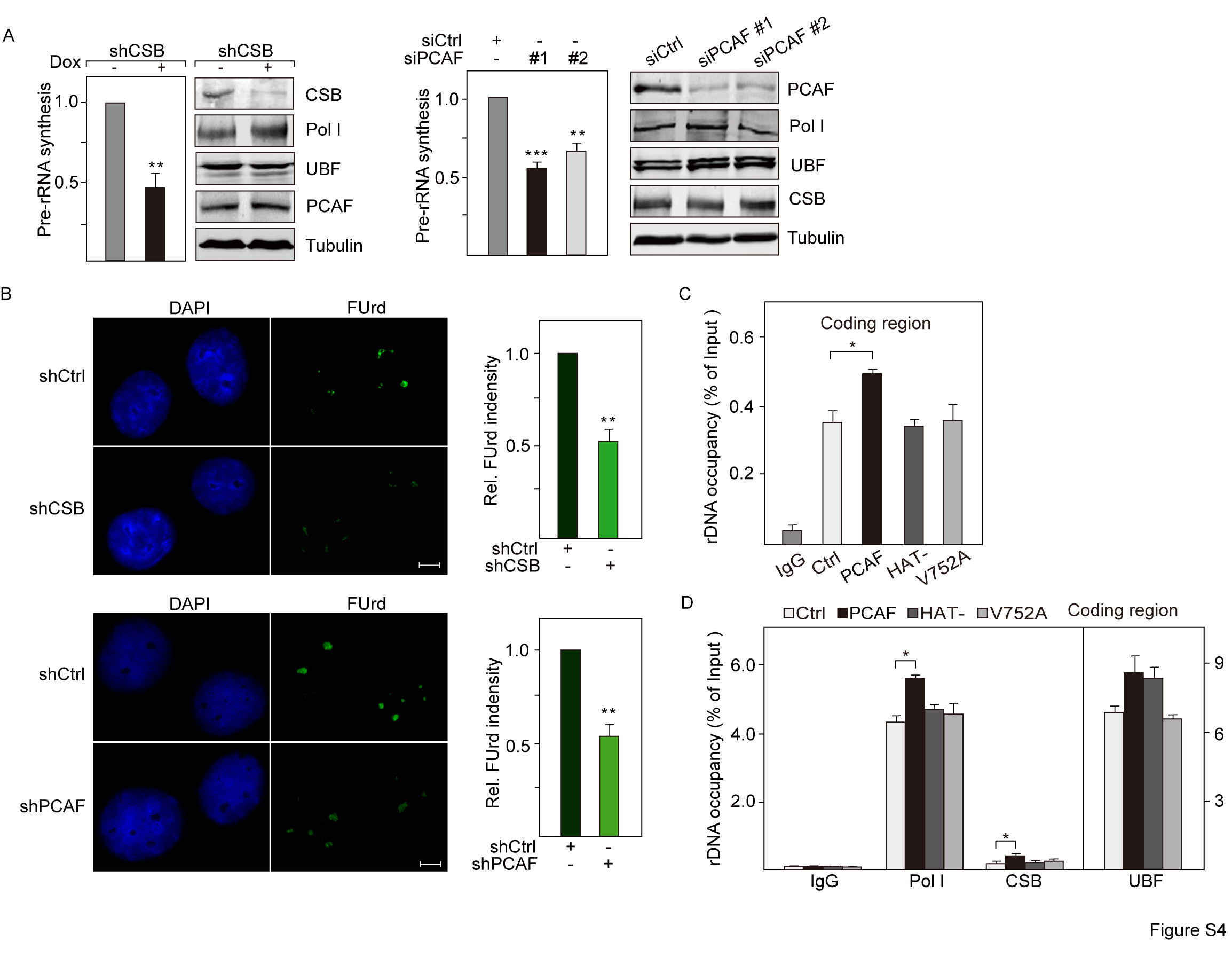

Supplement: Figure S4 — The association of PCAF with rDNA coding region requires HAT and bromodomain. A. Depletion of CSB or PCAF impairs 45S pre-rRNA synthesis. CSB was reduced in NIH 3T3 cells by tetracycline-induced synthesis of CSB-specific shRNA (shCSB). Cells were treated with 1 µg/ml doxycycline (dox) for 48 hr (left). On the right, 293T cells were transfected with PCAF-specific siRNAs (siPCAF #1 and #2) or control siRNA (siCtrl). 45S pre-rRNA synthesis was measured by qRT-PCR and normalized to GAPDH mRNA (n = 3). **P value <0.01, ***P value <0.001. Knockdown of CSB or PCAF was determined by western blot analysis using antibodies against Pol I, UBF, and tubulin. B. Knockdown of CSB or PCAF impairs nucleolar transcription. NIH 3T3 cells with knockdown of CSB (shCSB) or PCAF (shPCAF) were labeled for 15 min with fluorouridine (FUrd), and stained with antibodies to BrdU. Bar diagram represents intensity of FUrd labeling signals. Scale bar, 5 µm. **P value <0.01. C. PCAF mutants do not bind to rDNA coding region. ChIP data show the occupancy of PCAF and PCAF mutants on rDNA coding region in NIH 3T3 cells overexpressing wildtype PCAF, HAT-deficient mutant or bromodomain mutant. The immunoprecipitated DNA from PCAF overexpressed cells and mock-transfected cells (Ctrl) was normalized to input DNA. The levels of indicated proteins are shown with the standard deviation from three independent experiments. *P value <0.05. D. Overexpression of PCAF increases the occupancy of Pol I and CSB on rDNA coding region. ChIP data show the occupancy of UBF, Pol I and CSB on rDNA coding region in NIH 3T3 cells overexpressing wildtype PCAF, HAT-deficient mutant or bromodomain mutant. The levels of indicated proteins from PCAF overexpressed cells and mock-transfected cells (Ctrl) were normalized to input DNA (n = 3). *P value <0.05. (TIF) [file pone.0062668.s004.tif]

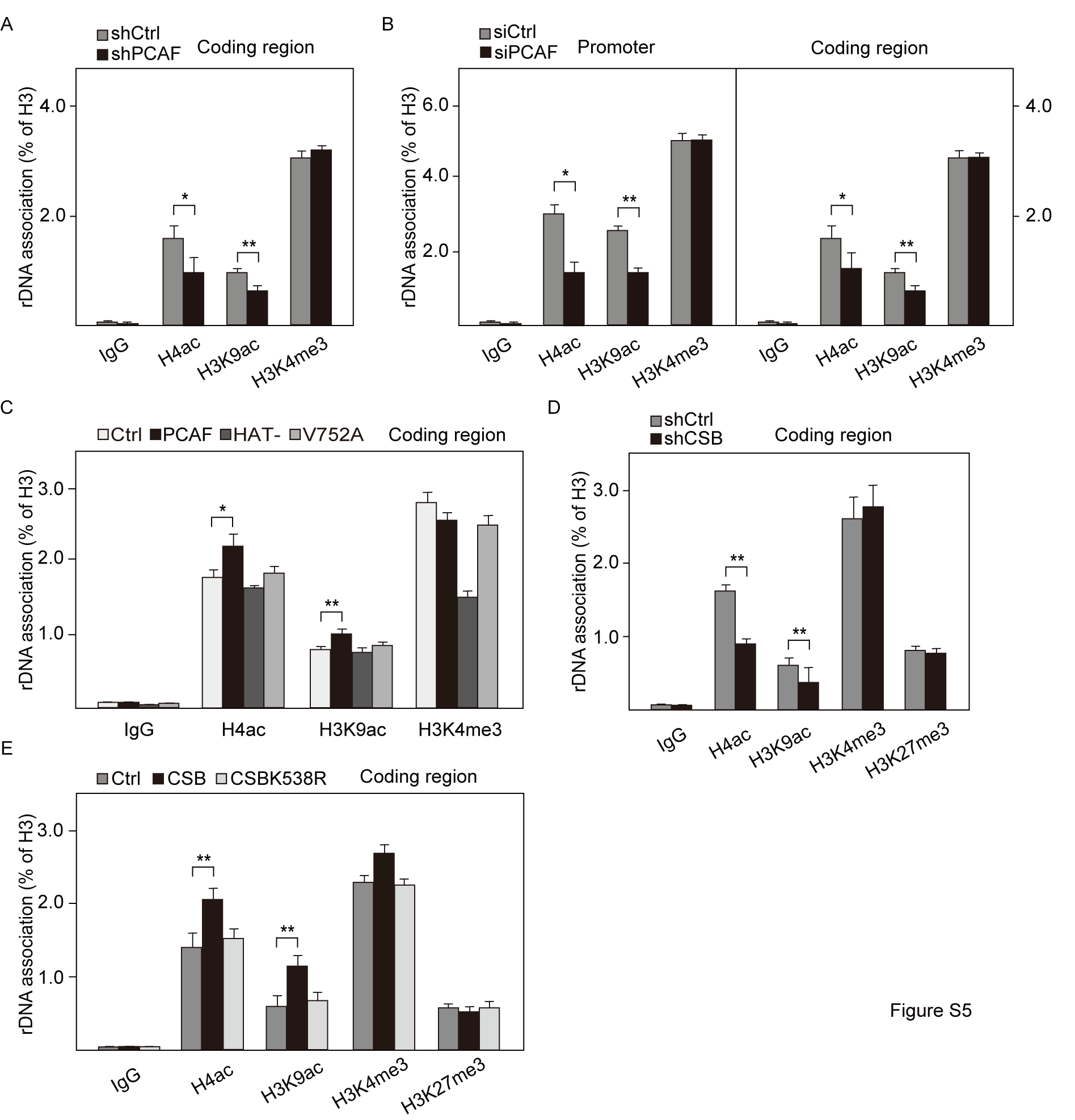

Supplement: Figure S5 — PCAF stimulates rRNA gene transcription by correlating with histone acetylation at the coding region. A. Depletion of PCAF decreases the binding of H4ac and H3K9ac to the rDNA coding region. ChIP data show the associations of H4ac, H3K9ac and H3K4me3 with rDNA coding region in NIH 3T3 cells infected with lentiviruses encoding PCAF-specific shRNA (black bars) or control shRNA (gray bars). Values of the average %IP (±standard deviation) for histone modifications from PCAF knockdown cells (shPCAF) and control cells (shCtrl) were normalized to histone H3. Error bars represent standard deviation (n = 3). *P value <0.05, **P value <0.01. B. Knockdown of PCAF decreases the binding of H4ac and H3K9ac to the rDNA. ChIP data were from 293T cells after siRNA-mediated depletion of PCAF. The levels of histone modifications from PCAF knockdown cells (siPCAF) and control cells (siCtrl) were normalized to histone H3. Error bars represent standard deviation (n = 3). *P value <0.05, **P value <0.01. C. Overexpression of PCAF promotes the binding of H4ac and H3K9ac to the rDNA coding region. Data from ChIP experiments show the rDNA occupancy of H4ac, H3K9ac and H3K4me3 in NIH 3T3 cells overexpressing wildtype PCAF and two mutants. The levels of the average %IP (±standard deviation) for H4ac, H3K9ac and H3K4me3 from PCAF overexpressed cells and mock-transfected cells (Ctrl) were normalized to histone H3. Error bars represent standard deviation (n = 3). *P value <0.05, **P value <0.01. D. Depletion of CSB reduces the levels of histone H4 acetylation and histone H3K9 acetylation at rDNA coding region. Cross-linked chromatins from NIH 3T3 cells after shRNA-mediated depletion of CSB were immunoprecipitated with antibodies against H4ac, H3K9ac, H3K4me3 and H3K27me3. The levels of histone modifications from CSB knockdown cells (shCSB) and control cells (shCtrl) were normalized to histone H3. Error bars represent standard deviation (n = 3). **P value <0.01. E. Overexpression of CSB increases [file pone.0062668.s005.tif]
