# Supplementary material for: The Chromatin Remodeling Factor CSB Recruits Histone Acetyltransferase PCAF to rRNA Gene Promoters in Active State for Transcription Initiation
Source: PLoS One. 2013 May 7;8(5):e62668. doi: 10.1371/journal.pone.0062668 (PMC3646882; doi:10.1371/journal.pone.0062668)
Supplement: Table S1 — Primer lists. (DOCX) [file pone.0062668.s006.docx]

Supporting Table S1 Primer lists

| Name | Sequence | Usage |
| --- | --- | --- |
| hrDNA promoter  (-49 / +32) | Forward:5'-GGTATATCTTTCGCTCCGAG-3'  Reverse:5'-GACGACAGGTCGCCAGAGGA-3' | ChIP for rDNA promoter |
| hrDNA coding region  (+7936/+8036) | Forward:5'-GCGACCTCAGATCAGACGTGG-3'  Reverse:5'-CTGTTCACTCGCCGTTACTGAG-3' | ChIP for rDNA coding region |
| mrDNA IGS  (+37391/+37410, +37491/+37510) | Forward: 5’- AGGTCCTGGGACATATGCAG-3’  Reverse: 5’- CCAGGAGTGGTGTTTGTGTG-3’ | ChIP for rDNA IGS |
| mrDNA promoter  (-105/-88, -21/-1) | Forward: 5’-CCCAGGTATGACTTCCAG-3’  Reverse: 5’-ACCTATCTCCAGGTCCAATAG-3’ | ChIP for rDNA promoter |
| mrDNA18s coding  (+4020/+4037, +4134/+4152) | Forward: 5’-CTGCCAGGTAGCATATGC-3’  Reverse: 5’- CAAGTAGGAGAGGAGCGAG-3’ | ChIP for rDNA 18s coding region |
| mrDNA 28s coding  (+8124/+8144, +8203/+8224) | Forward: 5’-GCGACCTCAGATCAGACGTGG-3’  Reverse: 5’-CTGTTCACTCGCCGTTACTGAG-3’ | ChIP for rDNA 28s coding region |
| mPromoter  (-165/-146, -7/+16) | Forward: 5’-GACCAGTTGTTCCTTTGAGG-3’  Reverse:5’-AGGACAGCGTGTCAGTACCTATC-3’ | *Hpa* II digestion analysis of rDNA promoter |
| mCoding region  (+3548/3567, +3732/3751) | Forward: 5’- GTTGGTGTGGGGAGTGAATG-3’  Reverse:5’-CTCTTACCCGCTCTCCCCTC-3’ | *Hpa* II digestion analysis of rDNA coding region |
| mInternal control  (-105/-88, -7/+16) | Forward: 5’-CCCAGGTATGACTTCCAG-3’  Reverse:5’-AGGACAGCGTGTCAGTACCTATC-3’ | *Hpa* II digestion analysis of rDNA |
| Mouse pre-rRNA primer  (+598/+619, +745/+765) | Forward: 5’-CGTGTAAGACATTCCTATCTCG-3’  Reverse:5’-GCCCGCTGGCAGAACGAGAAG-3’ | RT-qPCR for 45S pre-rRNA |
| Human pre-rRNA primer  (+307/+325, +424/442) | Forward: 5’-GTCAGGCGTTCTCGTCTC-3’  Reverse:5’- GCACGACGTCACCACATC-3’ | RT-qPCR for 45S pre-rRNA |
| GAPDH | Forward: 5’-CCATCACCATGTTCCAGGAG-3’  Reverse: 5’-CCTGCTTCACCACCTTCTTG-3’ | RT-qPCR for GAPDH |
| mNB Pre-rRNA | Forward: 5’-ACTGACACGCTGTCCTTTCC-3’  Reverse: 5’- GGCTCCACCATTCCAACCG-3’ | Northern Blot for 45S pre-rRNA |
