# Supplementary material for: The Chromatin Remodeling Factor CSB Recruits Histone Acetyltransferase PCAF to rRNA Gene Promoters in Active State for Transcription Initiation
Source: PLoS One. 2013 May 7;8(5):e62668. doi: 10.1371/journal.pone.0062668 (PMC3646882; doi:10.1371/journal.pone.0062668)
Supplement: Table S2 — Values of the average % IP (± standard deviation) normalized to input DNA for three independent experiments are shown. (DOCX) [file pone.0062668.s007.docx]

Supporting Table S2 Values of the average % IP (± standard deviation) normalized to input DNA for three independent experiments are shown.

| Samples | IGS | Promoter | 18s coding region | 28s coding region |
| --- | --- | --- | --- | --- |
| IgG | 0.015 (±0.002) | 0.020 (±0.009) | 0.022 (±0.007) | 0.026 (±0.003) |
| PCAF | 0.120 (±0.028) | 0.205 (±0.009) | 0.290 (±0.046) | 0.345 (±0.035) |
| CSB | 0.125 (±0.007) | 0.190 (±0.061) | 0.200 (±0.052) | 0.240 (±0.021) |
| Pol I | 0.264 (±0.203) | 1.823 (±0.465) | 4.800 (±0.417) | 4.210 (±0.915) |
| TTF-1 | 0.120 (±0.150) | 2.310 (±0.102) | 0.140 (±0.131) | 0.210 (±0.120) |
