# Supplementary material for: The Chromatin Remodeling Factor CSB Recruits Histone Acetyltransferase PCAF to rRNA Gene Promoters in Active State for Transcription Initiation
Source: PLoS One. 2013 May 7;8(5):e62668. doi: 10.1371/journal.pone.0062668 (PMC3646882; doi:10.1371/journal.pone.0062668)
Supplement: Methods and References S1 — (DOC) [file pone.0062668.s008.doc]

**Supporting Methods and Supporting References**

**Methods S1**

**FUrd Labeling of Nascent RNA and Immunofluorescence**

NIH 3T3 cells were labeled for 15 min with 2 mM ﬂuorouridine (FUrd), permeabilized and ﬁxed with acetone for 10 min at −20°C. After washing with PBS and blocking with 1% BSA in PBS, cells were incubated with BrdU antibodies overnight at 4°C, and stained with secondary antibodies for 1 hr at room temperature.

**Immunoprecipitation**

Nuclear extracts from NIH 3T3 cells were incubated overnight with antibodies against PCAF at 4ºC. Co-precipitated proteins were detected on western blots.

**Knockdown of PCAF by siRNA**

Two siRNA directed against PCAF were synthesized using the following primer sets (Invitrogen). siPCAF #1 was synthesized using forward primer: 5’-CCACCAUGAGUGGUGUCUA (dTdT)-3’ and reverse primer: 5’-UAGACACCACUCAUGGUGG (dTdT)-3’ [1]. siPCAF #2 was made using forward primer: 5’-UCGCCGUGAAGAAAGCGCA (dTdT)-3’ and reverse primer: 5’-UGCGCUUUCUUCACGGCGA (dTdT)-3’ [2]. 293T cells were transfected with 50 nM siRNA using Lipofectamine 2000 Transfection Reagent (Invitrogen). After 48 hr, knockdown was determined by western blot analysis.

**References S2**

1. Linares LK, Kiernan R, Triboulet R, Chable-Bessia C, Latreille D, et al. (2007) Intrinsic ubiquitination activity of PCAF controls the stability of the oncoprotein Hdm2. Nat Cell Biol 9: 331-338.

2. Bres V, Tagami H, Peloponese JM, Loret E, Jeang KT, et al. (2002) Differential acetylation of Tat coordinates its interaction with the co-activators cyclin T1 and PCAF. EMBO J 21: 6811-6819.
